# Chikungunya virus iridocyclitis in Fuchs’ heterochromic iridocyclitis

**DOI:** 10.4103/0301-4738.71707

**Published:** 2010

**Authors:** Padmamalini Mahendradas, Rohit Shetty, J Malathi, H N Madhavan

**Affiliations:** Uveitis and Ocular Immunology Services, Narayana Nethralaya, Superspeciality Eye Hospital and Post Graduate Institute of Ophthalmology, 121/C, Chord Road, Rajajinagar, 1^st^ “R” Block, Bangalore - 560 010, Karnataka, India; 1 L and T Microbiology Research Centre, Vision Research Foundation, Sankara Nethralaya, 18, College Road, Chennai – 600 006, Tamil Nadu, India

**Keywords:** Chikungunya virus, Fuchs’ heterochromic iridocyclitis, real-time polymerase chain reaction

## Abstract

We are reporting a case of bilateral Fuchs’ heterochromic iridocyclitis with chikungunya virus infection in the left eye. A 20-year-old female was presented with a past history of fever suggestive of chikungunya with bilateral Fuchs’ heterochromic iridocyclitis and complicated cataract. She had a tripod dendritic pattern of keratic precipitates by confocal microscopy in the left eye with a stippled pattern of keratic precipitates in both eyes. The real-time polymerase chain reaction (RT-PCR) assay in the aqueous humor detected 98 copies/ml of chikungunya virus RNA. The patient underwent clear corneal phacoemulsification with in-the-bag intraocular lens implantation in the left eye with a good visual outcome. This is the first report where the presence of chikungunya virus RNA has been associated with a case of bilateral Fuchs’ heterochromic iridocyclitis.

Fuchs’ heterochromic iridocyclitis is a specific form of uveitis. Earlier studies suggest its association with infectious agents like toxoplasmosis,[1] toxocariasis,[2] rubella virus,[3] and herpes simplex virus[4]. We report a case of bilateral Fuchs’ heterochromic iridocyclitis with chikungunya virus infection identified by real-time polymerase chain reaction (RT-PCR) in the aqueous humor from the left eye.

## Case Report

**Figure 1:**
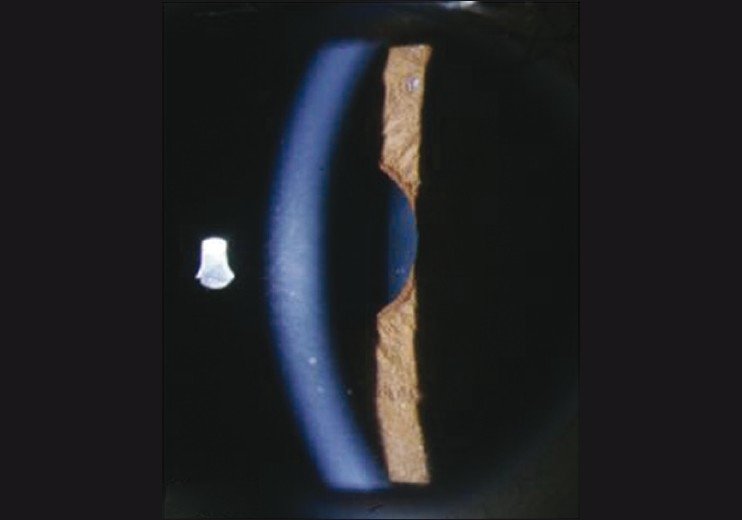
Slit-lamp biomicroscopic photograph of the right eye revealed fine-to-medium-sized keratic precipitates with heterochromia of the iris

**Figure 2:**
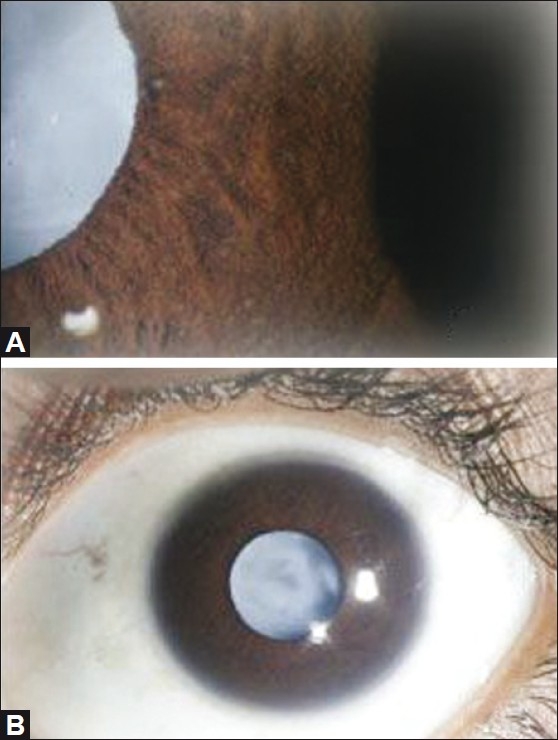
(A) Slit lamp biomicroscopic photograph revealed heterochromia of the iris, koeppe’s nodule with a mature cataract in the left eye. (B) Diffuse anterior segment photograph revealed quiet anterior segment, no posterior synechiae with mature cataract in the left eye.

**Figure 3:**
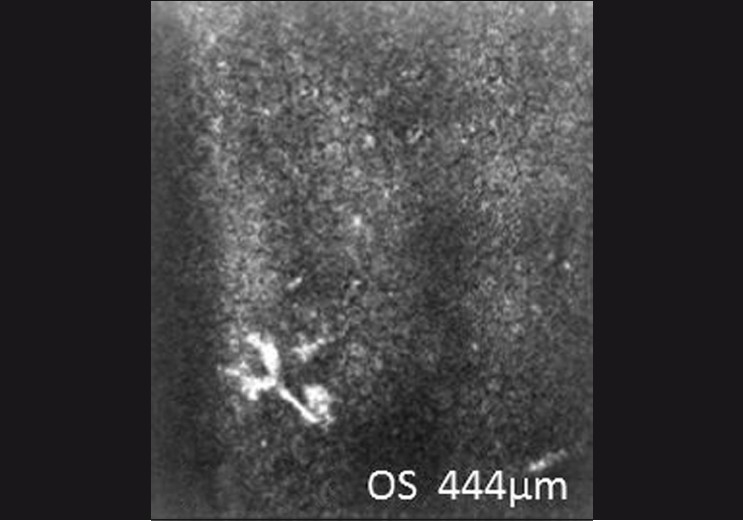
Confocal microscopy showing tripod dendritic with a stippled pattern of keratic precipitates in the left eye

A 20-year-old female of Indian origin presented with the complaint of decreased vision in the left eye since 2 weeks. One week prior to the onset of ocular symptoms, the patient had fever lasting for 3 days suggestive of chikungunya fever. Her ocular history was negative for trauma and previous inflammation. Her old investigational reports ordered by the physician revealed positive serum anti-rubella virus IgG (153 IU/ml), and serum toxoplasma, cytomegalovirus, and herpes simplex virus antibodies were negative. Her best corrected visual acuity was 20/30 in the right eye and counting finger close to face in the left eye. Intraocular pressure was normal in both the eyes. Slit-lamp biomicroscopy in the right eye demonstrated fine-to-medium-sized keratic precipitates, flare+, cells+, stromal atrophy of the iris [Fig. 1], nodules on the pupillary margin, and grade I posterior subcapsular opacity. The left eye revealed fine pigments on the corneal endothelium, flare+, cells+, small nodules on the iris [Fig. 2A], and shallow anterior chamber with a mature cataract in the left eye [Fig. 2B]. Gonioscopy revealed open angles in both eyes. Fundus examination of the right eye revealed vitritis+, vitreous haze+ with normal posterior segment; B-scan ultrasonography revealed few vitreous opacities with attached retina in the left eye. Confocal microscopy revealed a new tripod dendritic pattern with stippled keratic precipitates [Fig. 3] in the left eye with a stippled pattern of keratic precipitates in the right eye.

Diagnosis of bilateral Fuchs’ heterochromic iridocyclitis with a complicated cataract was made probably of viral origin. A sample of aqueous humor (AH) was drawn through a paracentesis at the beginning of the cataract surgery for RT-PCR and the anterior capsule was removed during the capsulorrhexis in the left eye for histopathological examination.

**Figure 4:**
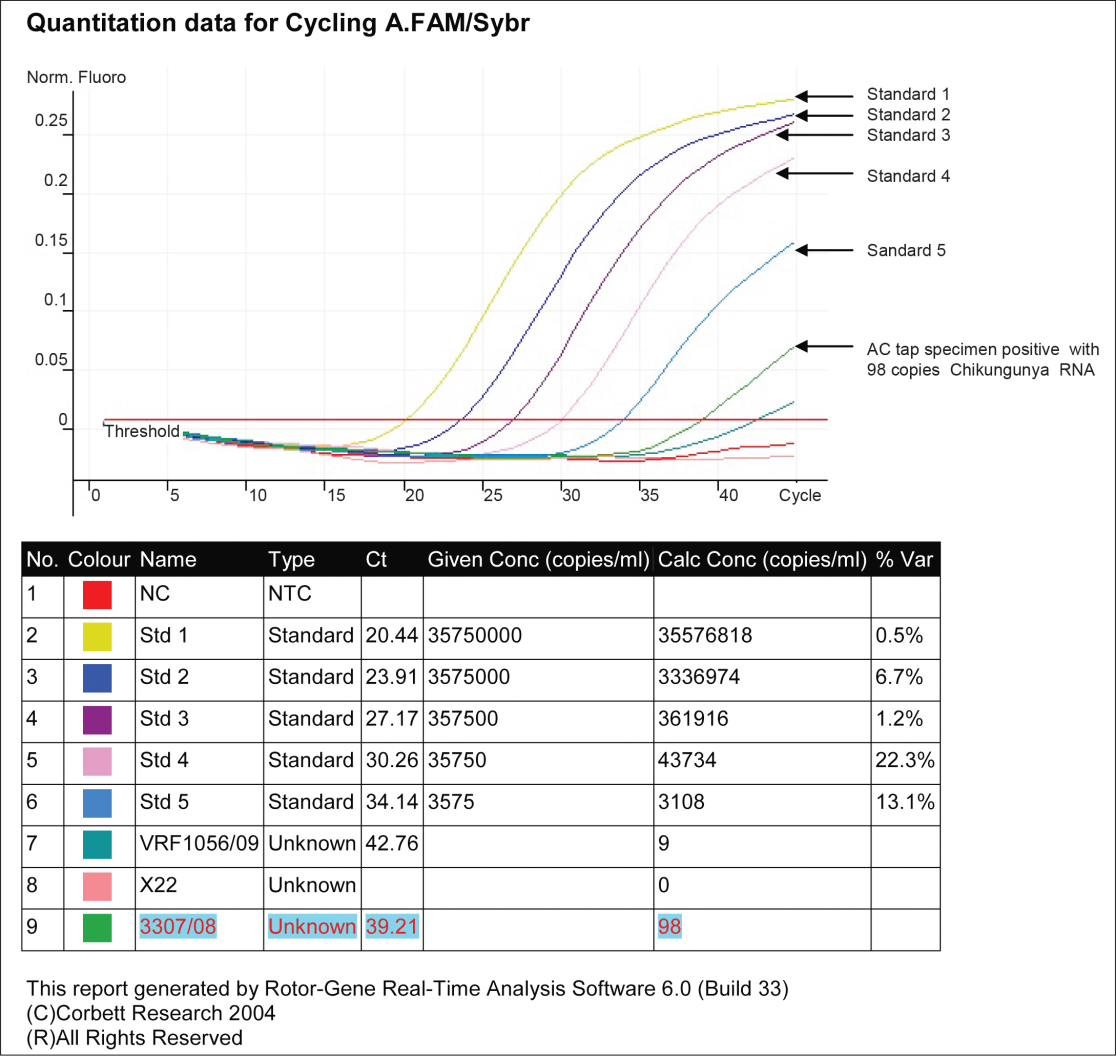
Kinetics of the real-time polymerase chain reaction performed with the anterior chamber tap collected from the left eye

**Figure 5:**
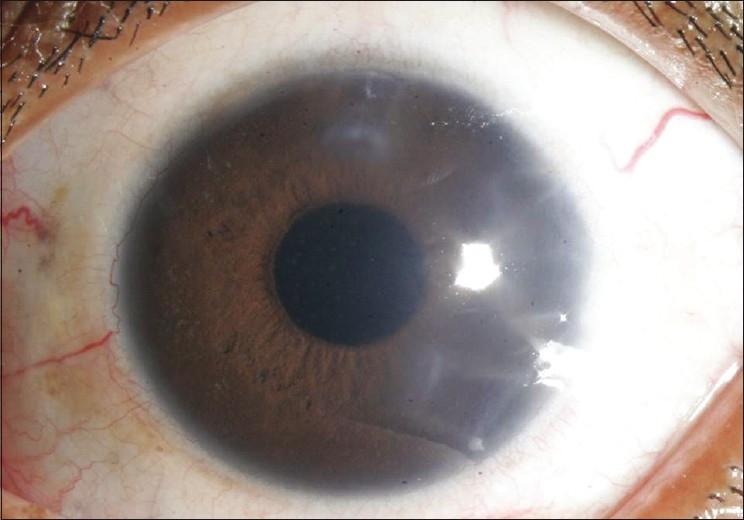
Diffuse anterior segment photograph of the first day postoperative picture in the left eye revealed pseudophakia

Reverse transcriptase PCR for rubella virus on the AH was carried out using primers and protocols as described earlier[5] found to be negative. Chikungunya virus was quantified in the AH using GenSens Chikungunya RT-PCR kit (Genome Diagnostics Pvt. Ltd., Solan, Himachal Pradesh, India) and Corbett Rotor Gene 6000 (Australia). The RT-PCR assay detected 98 copies/ml of chikungunya virus RNA in the anterior chamber tap [Fig. 4]. Lens capsule was negative for the microorganisms by histopathological examination. Following cataract surgery, her best corrected visual acuity improved to 20/20 in the left eye [Fig. 5].

## Discussion

Chikungunya is a single-stranded RNA virus. It can cause conjunctivitis, iridocyclitis,[6] episcleritis,[6] scleritis, keratitis, retinitis,[6,7] neuroretinitis,[8] choroiditis, panuveitis, and optic neuritis.

Confocal microscopy of keratic precipitates in Fuchs’ heterochromic iridocyclitis are globular keratic precipitates, infiltrating and dendritiform, stippled, and cruciform as described by Kanavi *et al*.[9]

The stippled pattern of keratic precipitates in both eyes was present which is similar to that reported by Kanavi *et al*. whereas the tripod dendritic pattern of keratic precipitates was noted in the left eye by confocal microscopy. We had an epidemic of chikungunya. The patient had fever suggestive of chikungunya which made us do the RT-PCR for chikungunya in the aqueous humor at the time of cataract surgery. The presence of chikungunya RNA virus in the AH of Fuchs’ heterochromic iridocyclitis suggests that chikungunya virus infection is present in a case of Fuchs’ heterochromic iridocyclitis, although it is not clear if the chikungunya virus was the initiator of the disease or a coincidental finding. This association reinforces the theory based on the putative role of an infectious agent associated with different forms of Fuchs’ heterochromic iridocyclitis. To the best of our knowledge, this is the first report where the presence of chikungunya virus RNA has been demonstrated in the case of Fuchs’ heterochromic iridocyclitis.

## References

[1] Toledo de Abreu M, Belfort R, Hirata PS (1982). Fuchs’ heterochromic cyclitis and ocular toxoplasmosis. Am J Ophthalmol.

[2] Teyssot N, Cassoux N, Lehoang P, Bodaghi B (2005). Fuchs’ heterochromic cyclitis and ocular toxocariasis. Am J Ophthalmol.

[3] Quentin CD, Reiber H (2004). Fuchs’ heterochromic cyclitis: Rubella virus antibodies and genome in aqueous humor. Am J Ophthalmol.

[4] Barequet IS, Li Q, Wang Y, O’Brien TP, Hooks JJ, Stark WJ (2000). Herpes simplex virus DNA identification from aqueous fluid in Fuchs heterochromic iridocyclitis. Am J Ophthalmol.

[5] Shyamala G, Malathi J, Moses YS, Therese KL, Madhavan HN (2007). Nested reverse transcription polymerase chain reaction for the detection of rubella virus in clinical specimens. Indian J Med Res.

[6] Mahendradas P, Ranganna SK, Shetty R, Balu R, Narayana KM, Babu RB (2007). Ocular Manifestations Associated with Chikungunya. Ophthalmology.

[7] Murthy KR, Venkataraman N, Satish V, Babu K (2008). Bilateral retinitis following chikungunya fever. Indian J Ophthalmol.

[8] Mahesh G, Giridhar A, Shedbele A, Kumar R, Saikumar SJ (2009). A case of bilateral presumed chikungunya neuroretinitis. Indian J Ophthalmol.

[9] Kanavi MR, Soheilian M, Yazdani S, Peyman GA (2010). Confocal scan features of keratic precipitates in Fuch’s heterochromic iridocyclitis. Cornea.

